# Development and evaluation of a multiplex droplet digital polymerase chain reaction method for simultaneous detection of five biothreat pathogens

**DOI:** 10.3389/fmicb.2022.970973

**Published:** 2022-07-28

**Authors:** Yipu Du, Ziheng Yan, Kai Song, Junyan Jin, Liting Xiao, Zhulin Sun, Yafang Tan, Pingping Zhang, Zongmin Du, Ruifu Yang, Yong Zhao, Yajun Song

**Affiliations:** ^1^State Key Laboratory of Pathogen and Biosecurity, Beijing Institute of Microbiology and Epidemiology, Academy of Military Medical Sciences (AMMS), Beijing, China; ^2^The First Department, General Hospital of Northern Theater Command, Shenyang, China; ^3^School of Basic Medical Sciences, Anhui Medical University, Hefei, China; ^4^College of Veterinary Medicine, South China Agricultural University, Guangzhou, China

**Keywords:** biothreat agent, molecular diagnosis, droplet digital PCR, multiplex detection, pathogen

## Abstract

Biothreat agents pose a huge threat to human and public health, necessitating the development of rapid and highly sensitive detection approaches. This study establishes a multiplex droplet digital polymerase chain reaction (ddPCR) method for simultaneously detecting five high-risk bacterial biothreats: *Yersinia pestis*, *Bacillus anthracis*, *Brucella* spp., *Burkholderia pseudomallei*, and *Francisella tularensis*. Unlike conventional multiplex real-time PCR (qPCR) methods, the multiplex ddPCR assay was developed using two types of probe fluorophores, allowing the assay to perform with a common two-color ddPCR system. After optimization, the assay performance was evaluated, showing a lower limit of detection (LOD) (0.1–1.0 pg/μL) and good selectivity for the five bacteria targets. The multiplex assay’s ability to simultaneously detect two or more kinds of targets in a sample was also demonstrated. The assay showed strong sample tolerance when testing simulated soil samples; the LOD for bacteria in soil was 2 × 10^2^–2 × 10^3^ colony-forming unit (CFU)/100 mg soil (around 5–50 CFU/reaction), which was 10-fold lower than that of the single-target qPCR method. When testing simulated soil samples at bacterial concentrations of 2 × 10^3^–2 × 10^4^ CFU/100 mg soil, the assay presented a higher sensitivity (100%, 35/35) than that of the qPCR method (65.71%, 23/35) and a good specificity (100%, 15/15). These results suggest that the developed 5-plex ddPCR method is more sensitive than conventional qPCR methods and is potentially suitable for rapidly detecting or screening the five selected bacterial biothreats in suspicious samples.

## Introduction

Biothreat agents pose a significant threat to global public health and security because they are easily transmitted and cause high mortality rates. According to the United States Centre for Disease Control and Prevention (CDC), *Yersinia pestis*, *Bacillus anthracis*, and *Francisella tularensis* are classified as category A agents among the potential biothreat agents, and *Brucella* spp. and *Burkholderia pseudomallei* are classified as category B agents. These five bacterial pathogens could cause serious human infectious diseases such as plague, anthrax, brucellosis, melioidosis, and tularemia (Barras and Greub, 2014; Oliveira et al., 2020; Long and Marzi, 2021). For early response and control of bioterrorism incidents, rapid and accurate detection of these bacteria biothreats from suspicious samples is critical. However, conventional culture-based bacterial detection methods require several days, which calls for a more rapid approach (Ricchi et al., 2017). Therefore, various diagnostic techniques, especially polymerase chain reaction (PCR) based methods, have been developed to rapidly detect biothreat pathogens (Yang and Rothman, 2004; Yeh et al., 2019).

Droplet digital PCR (ddPCR) is a new PCR technology that can achieve sensitive and accurate quantification of target molecules without using quantitative curves (Vogelstein and Kinzler, 1999; Hindson et al., 2013). It has excellent performance for absolute quantification of low-level targets and robust resistance to various inhibitors compared with traditional real-time PCR (qPCR), making it particularly ideal for the detection of pathogens in complex matrices (soil, powder, foods, etc.) (Dong et al., 2020; Basanisi et al., 2022; Costa et al., 2022). Several studies have shown that ddPCR may be used to detect infectious bacteria or viruses with great sensitivity (Lei et al., 2021; Vasudevan et al., 2021); however, few were focused on the biothreat agents (Straub et al., 2013; Ricchi et al., 2017). Moreover, there are relatively fewer multiplex ddPCR assays than multiplex qPCR assays since the most common ddPCR systems only have two fluorescent channels, limiting the multiplex detection capacity.

**FIGURE 1:**
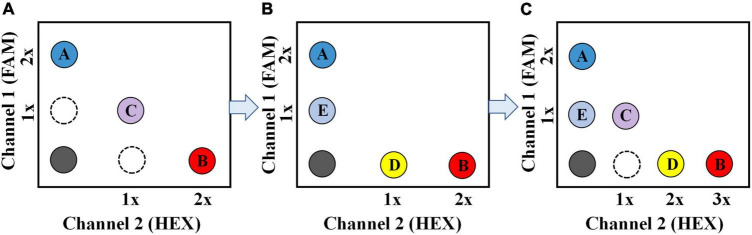
Schematic description of the multiplex ddPCR assay for simultaneous detection of three targets **(A)**, four targets **(B)**, and five targets **(C)**, respectively. For the 5-plex ddPCR assay, target A and B are recognized by a FAM -labeled (2 × conc.) and a HEX-labeled probe (3 × conc.), respectively; Target C is recognized by a FAM-labeled (1 × conc.) probe and a HEX-labeled probe (1 × conc.) simultaneously; target D and E are recognized by a HEX-labeled (2 × conc.) and a FAM-labeled probe (1 × conc.), respectively.

Researchers have developed several multiplex ddPCR methods with different strategies (Lei et al., 2020; Leong et al., 2020; Nyaruaba et al., 2021) based on common two-color ddPCR systems. (1) One representative strategy is based on the probe-mixing multiplexing approach (Figure 1A). When a target gene or fragment is simultaneously detected by two fluorescent probes: one is labeled with the fluorophore of 6-Carboxyfluorescein (FAM), another one is labeled with 5′-Hexachlorofluorescein (HEX), the amplification will generate two-color combined fluorescent signals, which could be distinguished from those single-color signals by the ddPCR. In this way, Vicky Rowlands et al. (2019) developed a multiplex ddPCR assay to detect four point mutations in the human PIK3CA gene simultaneously; one of the point mutations was analyzed using two probes: one labeled with FAM (375 nM), another one labeled with HEX (250 nM). (2) Another representative strategy uses the amplitude-based multiplexing method (Figure 1B). Two different targeted probes with the same fluorophore can also be distinguished in the condition that the two probes maintain a sufficient concentration difference in the ddPCR reaction mixture. This difference in probe concentration will produce a difference in fluorescence amplitude, allowing the two probes to be distinguished. Using this approach, Lei et al. (2020) developed a 4-plex ddPCR assay to simultaneously detect four target genes of *Vibrio parahaemolyticus* in food samples. The probes for *tlh* (125 nM) and *ureR* gene (250 nM) were labeled with FAM, and the probes for *tdh* (625 nM) and *orf8* gene (1250 nM) were labeled with another fluorophore. The four target genes could be clearly identified based on fluorescence amplitude due to the reasonable proration of these probes. Most multiplex ddPCR assays were developed using the two abovementioned approaches. In our perspective, the multiplex detection capacity could be further improved by combining the two approaches and applying them for the multiplex detection of biothreat pathogens (Figure 1C).

In the study, we developed a 5-plex ddPCR-based method for highly sensitive and simultaneous detection of five selected pathogens: *Y. pestis*, *B. pseudomallei*, *B. anthracis*, *Brucella* spp., and *F. tularensis*, with a two-color ddPCR system (Bio-Rad QX200™). Performances of the assay were comprehensively evaluated compared with conventional single-target ddPCR and qPCR methods. Its ability to detect single and multiple target pathogens in real soil samples was also tested, showing its suitability for the rapid and sensitive biothreat pathogens detection in suspicious soil samples.

## Materials and methods

### Bacteria culture and deoxyribonucleic acid extraction

All bacteria strains used in the study were preserved in the laboratory of Beijing Institute of Microbiology and Epidemiology (Beijing, China). All experiments involving bacterial culture were approved and carried out in the laboratory under qualified biosafety conditions. *Y. pestis* strain 201 (CBSLAM 1974), *B. anthracis* stern strain (CBSLAM 00067), *B. pseudomallei* (ATCC 23343), and *B. abortus* (CBSLAM 6148) were grown on the LB (Luria-Bertani) medium; *F. tularensis* (CBSLAM 6339) was grown on the BHI (Brain Heart Infusion) medium. Bacteria solutions were cultured overnight at 37°C with shaking at 200 rpm, and then the bacteria number was determined using the plate-counting method. Deoxyribonucleic acid (DNA) was extracted with a QIAamp^®^ DNA Mini Kit (Qiagen 51304, Germany). NanoDrop One (Thermo Fisher Scientific, United States) was used to determine the DNA concentration.

### Primers and probes

Table 1 shows the list of all primers and TaqMan^®^ probes used in the study. According to previous reports (Wang et al., 2006; Shi et al., 2009; Qu et al., 2010), to detect *F. tularensis*, *B. anthracis*, and *Y. pestis*, respectively, three sets of specific primers and probes were used. Among them, *B. anthracis* was detected with two probes, one labeled with FAM and another labeled with HEX. Another two sets of primers and probes for *B. pseudomallei* and *Brucella* spp. were developed in this study. All the primers and probes were anchored to the chromosomal genes, considering the possibility of plasmid gene loss in bacteria. All the primers and probes were synthesized by the Sangon Biotech Company (Shanghai, China).

**TABLE 1:** Primer and probe sequences for the five bacteria targets.

Bacteria	Target gene	*^a^*Sequence 5′–3′	References
***Y. pestis***	*3a*	F: GGACGGCATCACGATTCTCT R: CCTGAAAACTTGGCAGCAGTT P: FAM-CCCTCGAATCGCTGGCCAACTG-BHQ1	Qu et al., 2010
***B. pseudomallei***	*^b^DP58_RS29725*	F: CGATCTCGTCAAGGTGTCGG R: TTGACCTGGATGGCAAAGAAG P: FAM-TTGCCTCAGTCACGCGCACGT-BHQ1	This study
***B. anthracis***	*gs*	F: GGGTGTAATGTGAAGTAACTCGCTA R: AAACCGCTGTAAGAATGGAATTACG P1: FAM-CGTTGTAACATCGGCTTAGAGAACCACA-BHQ1 P2: HEX-CGTTGTAACATCGGCTTAGAGAACCACA-BHQ1	Wang et al., 2006
***Brucella*** **spp.**	*bp26*	F: TCAGGGCGGTGATTTGAAC R: GCGCGCCTCGTTGATC P: HEX-TGGTCAATGATAATCCCTCCGCCG-BHQ1	This study
***F. tularensis***	*AKR*	F: GCAGGGCGAGCACCATT R: ATCTTGCATGGTCACCACTTGA P: HEX-CGATATTTGCCTGTTAGCACTCCT-BHQ1	Shi et al., 2009

^a^F, forward primer; R, reverse primer; P, Taqman probe; BHQ, black hole quencher.^b^Gene locus corresponding to the reference genome (NZ_CP008782.1) of B. pseudomallei.

### Real-time polymerase chain reaction

The qPCR mixture comprised 10 μL Luna Universal qPCR Probe Master Mix (Roche Diagnostics GmbH, Germany), 400 nM primers, 200 nM probes, 2 μL DNA sample, and DNAase-free water to a final volume of 20 μL. Thermal cycling was set to run for 5 min at 95°C to activate the enzyme, followed by 40 cycles of denaturation at 95°C for 10 s and annealing at 60°C for 30 s (fluorescence collections). All qPCR reactions were performed with a CFX Opus 96 Real-Time PCR System (Bio-Rad, United States).

### Droplet digital polymerase chain reaction

Each single-target ddPCR reaction mixture for the five targets consisted of 10 μL 1 × ddPCR Supermix for probes (Bio-Rad, United States), 900 nM specific primers, 250 nM probes, 2.0 μL DNA, and DNAase-free water to a final volume of 20 μL.

The 5-plex ddPCR reaction mixture consisted of 10 μL 1 × ddPCR Supermix for probes (Bio-Rad, United States), 900 nM primers for each target, 250 nM *B. pseudomallei* probe (FAM-labeled), 500 nM *Y. pestis* probe (FAM-labeled), 500 nM *Brucella* spp. probe (HEX-labeled), 750 nM *F. tularensis* probe (HEX-labeled), 250 nM *B. anthracis* probe-1 (FAM-labeled), 250 nM *B. anthracis* probe-2 (HEX-labeled), 2 μL DNA, and DNAase-free water to a final volume of 20 μL.

All ddPCR assays were performed using a two-color Bio-Rad QX200™ ddPCR system. The QX200™ system consists of a droplet generator and an automated reader. The ddPCR reaction mixture was first transferred to the droplet generator, generating up to 20,000 nanoliter-sized droplets, and then loaded into a T100™ Thermal Cycler (Bio-Rad, United States) for amplification. Thermal cycling was set to run for 10 min at 95°C with a ramp rate of 2°C/s at each step; followed by 40 cycles of denaturation at 94°C for 30 s and 1 min annealing/extension at 60°C; enzyme deactivation at 98°C for 10 min; and a final step at 12°C for at least 30 min to stabilize the droplets. After amplification, the droplets were read in the QX200™ reader, and the data were analyzed using the Bio-Rad QuantaSoft™ Analysis Pro software.

### Sensitivity and quantification strategy of the multiplex droplet digital polymerase chain reaction

Bacteria DNA was diluted in DNAase-free water from 1.0 ng/μL to 1.0 fg/μL. Each sample was tested in triplicate using the multiplex ddPCR assay, as well as the single-target ddPCR assay and qPCR assay. According to the NCCLS guideline of EP17-A (Moretti et al., 2011), another 30 DNAase-free water samples were tested as blank samples to determine the limit of blank (LOB) for each channel.

LOB = Result at position [*N*_*B*_ (*p*/100) +0.5] = Result at position [0.95 × *N*_*B*_+0.5] (1)

Where *N*_*B*_ is the total number of blank samples, *p* is the proportion of assignment of the experimental data in descending order, and *p* is 95% in this study.

Samples with copy numbers above the LOB were considered positive. The lowest DNA concentration that could be detected was determined as the limit of detection (LOD). The quantitative curves for each target were constructed with log10 (DNA concentration, pg/μL) as the *x*-axis and log10 (copies/reaction by ddPCR) as the *y*-axis, respectively. The linear fitting coefficient (R2) was calculated with Origin 8.0.

### Specificity of the multiplex droplet digital polymerase chain reaction

The specificity of the assay was evaluated with a total of 25 other pathogenic bacteria, including 9 species related to *Y. pestis* (*Yersinia enterocolitica*, *Yersinia pseudotuberculosis*, *Yersinia rohdei*, *Yersinia mollaretii*, *Yersinia kristensenii*, *Yersinia bercovieri*, *Yersinia aldovae*, *Yersinia ruckeri*, *Yersinia frederiksenii*), 7 species related to *B. anthracis* (*Bacillus cereus*, *Bacillus thuringiensis*, *Bacillus subtilis*, *Bacillus megaterium*, *Bacillus pumilus*, *Clostridium difficile*, *Pseudomonas aeruginosa*), 5 species related to *B. pseudomallei* (*Burkholderia mallei*, *Burkholderia gladioli*, *Burkholderia cepacia*, *Burkholderia thailandensis*, *Burkholderia vietnamiensis*), and 4 other common pathogens (*Staphylococcus aureus*, *Salmonella enteritidis*, *Shigella dysenteriae*, *Escherichia coli*). DNA samples were diluted to 1.0 ng/μL as the ddPCR template. Each DNA was tested three independent times.

### Simultaneous detection of samples with multiple targets

A total of 26 DNA samples mixed with at least two target DNA were prepared, including 10 combinations ($C_{5}^{2}$) of two-target mixture, 10 combinations ($C_{5}^{3}$) of the three-target mixture, 5 combinations ($C_{5}^{1}$) of the four-target mixture, and 1 combination of the five-target mixture. The concentration of each target DNA in the mixed samples was 0.01–0.1 ng/μL. The 5-plex ddPCR assay was used to detect these samples as described above. According to the area and fluorescence intensity of the droplets in the two-dimensional fluorescence scatter plot (2D plot), the positive droplets generated by the mixed samples can be divided into corresponding target areas and counted separately. All tests were repeated three times.

### Evaluation of the droplet digital polymerase chain reaction method with spiked soil samples

Standard bacteria solutions were prepared at concentrations from 2 × 10^2^ to 2 × 10^8^ colony-forming unit (CFU)/mL. After centrifuging 1 mL of bacterial solution at 8000 rpm for 10 min, 900 μL of the supernatant was discarded. The remaining 100 μL were mixed with 100 mg of soil from a local garden. The spiked soil samples were then incubated at room temperature for 12 h. In addition, another 30 soil samples were spiked with 100 μL DNAase-free water as blank samples to determine the LOBs for soil samples according to the above NCCLS guideline of EP17-A. Then, all the samples were extracted with a TIANamp Soil DNA Kit (TianGen, Beijing, China). The extracted DNA was finally eluted with 80 μL of DNAase-free water; 2 μL of the DNA was used as the template of the multiplex ddPCR.

Furthermore, 50 simulated soil samples, including 15 negative samples and 35 positive samples, were prepared to evaluate the detection accuracy of the assay. Among the positive samples, 17 were spiked with one single kind of the target, 8 were spiked with two kinds of the targets, and 10 were spiked with three kinds of the targets (Supplementary Table 1). The concentration of each bacteria target in the soil samples was 2 × 10^3^–2 × 10^4^ CFU/100 mg soil. Genome DNA from these soil samples was extracted with the TIANamp Soil DNA Kit. All the samples were tested by the multiplex ddPCR and single-target qPCR assay, respectively.

## Results

### Establishment of the 5-plex droplet digital polymerase chain reaction method

**FIGURE 2:**
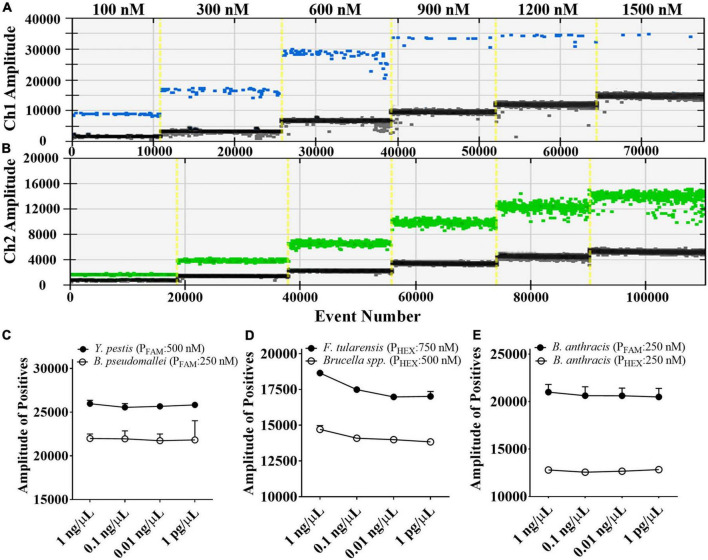
Fluorescence amplitude of ddPCR mixtures containing different concentrations of FAM-labeled probes **(A)** and HEX-labeled probes **(B)**, and the fluorescence amplitude corresponding to serial diluted DNA samples (1 pg/μL to 1 ng/μL) under the optimized probe concentrations **(C–E)**.

We tested the dose-response relationship between probe concentrations and fluorescence amplitudes by first developing a single-target ddPCR assay for each target. It was discovered that as the concentration of FAM-labeled or HEX-labeled probes was increased, the droplet fluorescence intensity gradually increased until the probe concentration reached about 900 and 1200 nM, respectively (Figures 2A,B). Therefore, with a reasonable ratio of probe concentrations, the probes labeled with the same fluorophore can be distinguished by the fluorescence amplitude. On this basis, in the proposed 5-plex ddPCR assay, we set the concentrations of FAM-labeled probes for *B. pseudomallei* and *Y. pestis* to 250 and 500 nM, respectively (Figure 2C); and set the concentrations of HEX-labeled probes for *Brucella* spp. and *F. tularensis* to 500 and 750 nM, respectively (Figure 2D). These four targets could be well differentiated using the fluorescence channel and amplitude, even at different template concentrations (1 pg/μL–1 ng/μL).

**FIGURE 3:**
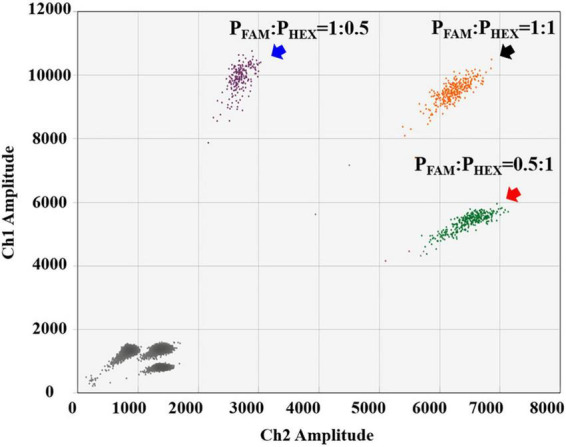
Comparison of different probe-concentration ratios (FAM-labeled and HEX-labeled probes) for the detection of *B. anthracis*.

For the detection of *B. anthracis*, we used the probe-mixing approach: one part of the probes was labeled with FAM (250 nM), and the other part was labeled with HEX (250 nM) (Figure 2E). In this way, the *B. anthracis* amplification could generate positive signals in both fluorescent channels, which can be distinguished from the other four targets in the 2D plot. The droplet clusters corresponding to *B. anthracis* had better spatial discrimination in the 2D plot when the concentration ratio of the two probes was 1:1 (Figure 3). We successfully established a 5-plex ddPCR assay for the five biothreat targets with a dual-fluorescence channel ddPCR instrument by combining the amplitude-based multiplexing and probe-mixing multiplexing approaches.

### Evaluation of the 5-plex droplet digital polymerase chain reaction method with deoxyribonucleic acid samples

**FIGURE 4:**
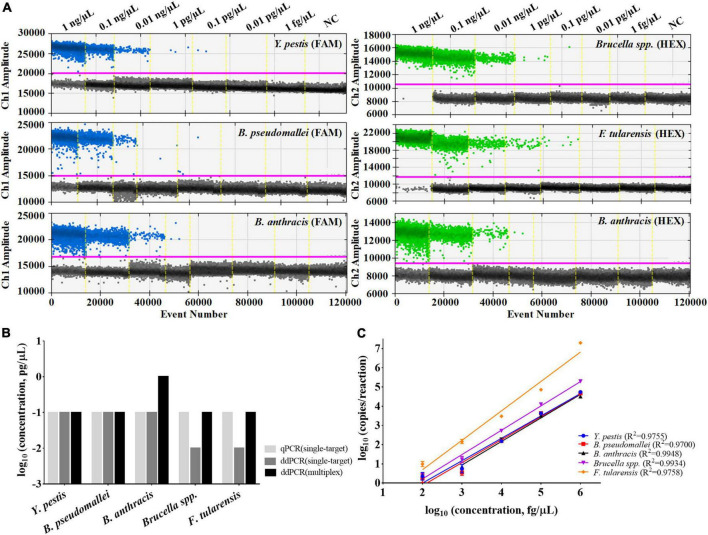
Performances of the multiplex ddPCR assay. **(A)** Detection results of the target DNA with concentrations from 1 fg/μL to 1 ng/μL by the multiplex assay. **(B)** Comparisons of the LODs between single-target qPCR, single-target ddPCR and the multiplex ddPCR method. **(C)** Quantification linearity of the target DNA by multiplex ddPCR. The purple line indicates the threshold for positive signals. NC refers to the negative control (DNAase-free water).

The multiplex ddPCR assay’s detection sensitivity for each target was evaluated by testing a series of diluted DNA solutions (1 fg/μL–1 ng/μL). The results showed that the LODs for *Y. pestis*, *B. pseudomallei*, *Brucella* spp., and *F. tularensis* all reached 0.1 pg/μL; with the exception of *B. anthracis*, which was 1.0 pg/μL (Figure 4A). For *Y. pestis* and *B. pseudomallei*, the LODs of the multiplex assay were comparable to those of the single-target ddPCR assays; for *Brucella* spp., *F. tularensis*, and *B. anthracis*, the LODs of the multiplex assay were ten-fold higher (Figure 4B). This slight decrease in sensitivity might be mainly due to the component complexity of the multiplex assay. However, the sensitivity of the multiplex ddPCR assay was generally consistent with that of the single-target qPCR assay, suggesting a high sensitivity of the developed assay.

The quantitative curves for the five DNA targets were also established, with log_10_ (concentrations of the target DNA template, fg/μL) on the *x*-axis and log_10_ (copies/reaction by the multiplex ddPCR) on the *y*-axis (Figure 4C). For each target, it shows high quantitative linearity (*R*^2^≥ 0.9700) at concentrations ranging from 1 pg/μL to 1 ng/μL (for *B. anthracis*) or 0.1 pg/μL to 1 ng/μL (for the other four targets). For DNA templates of high concentrations (≥10 ng/μL), only qualitative analysis could be performed because the number of generated droplets has reached the limit of the instrument used in the study (around 20,000).

A total of 25 other closely related bacteria species and pathogens were used to test the assay specificity, including *Y. enterocolitica*, *Y. pseudotuberculosis*, *Y. rohdei*, *Y. mollaretii*, *Y. kristensenii*, *Y. bercovieri*, *Y. aldovae*, *Y. ruckeri*, *Y. frederiksenii*, *B. cereus*, *B. thuringiensis*, *B. subtilis*, *B. megaterium*, *B. pumilus*, *C. difficile*, *P. aeruginosa*, *B. mallei*, *B. gladioli*, *B. cepacia*, *B. thailandensis*, *B. vietnamiensis*, *S. aureus*, *S. enteritidis*, *S. dysenteriae*, and *E. coli*. As shown in Supplementary Figure 1, there is no cross-amplification for these bacterial DNA (1 ng/μL), which indicated the specificity of our multiplex assay.

### Simultaneous detection of multiple targets by droplet digital polymerase chain reaction

**FIGURE 5:**
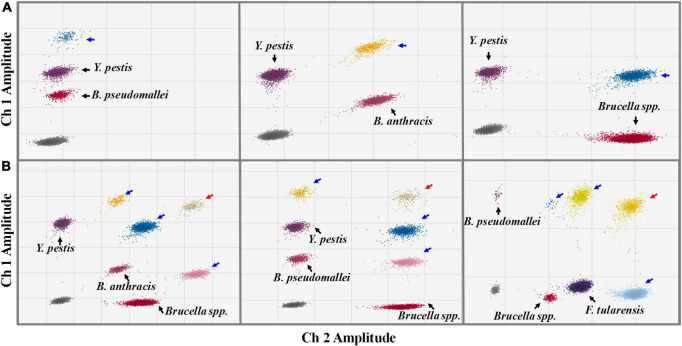
Simultaneous detections of two **(A)** and three **(B)** kinds of targets in one sample by the ddPCR assay (2D plot). The *x*-axis refers to fluorescence amplitude in the FAM channel; the *y*-axis refers to fluorescence amplitude in the HEX channel. The droplet clusters marked by black, blue, and red arrows refer to positive droplets containing a single target, two different targets, and three different kinds of targets, respectively.

We also investigated the assay’s ability to detect two or more DNA targets simultaneously, considering the possibility of multiple targets in one suspicious sample. When two different targets were present in the sample, the ddPCR generated four droplet clusters on the 2D plot: one cluster corresponding to the negative droplets, two clusters corresponding to the droplets containing one single target, and one additional cluster (Figure 5A). The additional cluster represents the positive droplets simultaneously containing both two kinds of targets; the superimposed fluorescence signal results in enhanced or shifted signals. When three or more targets were present in one sample, the number of additional clusters would increase substantially (Figure 5B). According to the fluorescence intensity and the region in which they are located, each new cluster on the 2D plot can be interpreted in terms of which kind of target it contains.

### Sample tolerance to real soil samples

**FIGURE 6:**
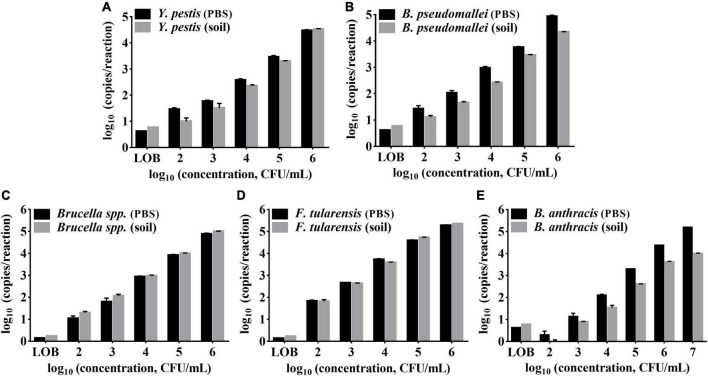
Performances of the multiplex ddPCR assay detecting target bacteria in PBS solutions and soil samples. **(A–E)** Represents the target of *Y. pestis*, *B. pseudomallei*, *B. anthracis*, *Brucella spp*., and *F. tularensis*,respectively. The *x*-axis refers to log_10_ (ddPCR results, copies/reaction); the *y*-axis refers to log_10_ (bacteria concentration, CFU/mL). Each test was repeated in triplets. LOBs were determined to be 5.905 copies/reaction and 1.725 copies/reaction for the FAM and HEX channels, respectively.

Soil, for example, is a common type of environmental sample that contains PCR inhibitors such as humic acid (Sidstedt et al., 2020). To evaluate the assay tolerance to soil matrices, bacteria sediment from 1 mL of phosphate buffer saline (PBS) (2 × 10^2^–2 × 10^8^ CFU/mL) was added to 100 mg of soil to prepare the simulated soil samples and tested by the ddPCR method. As shown in Figure 6, the detection results of the soil samples were compared with those of the bacteria in PBS. The results (copies/reaction) of soil samples were lower than those of solution samples for *Y. pestis*, *B. pseudomallei*, *Brucella* spp., and *B. anthracis*. However, the LODs of those strains in soils were consistent with the LODs for pure bacteria solutions, demonstrating the suitability of the assay to detect targets in soil. The LODs for *Y. pestis*, *B. pseudomallei*, *Brucella* spp., and *F. tularensis* all reached 2 × 10^2^ CFU/mL (appropriately 5 CFU/reaction, as only 2 μL of DNA from 80 μL of the extracted DNA was added to the reaction, and the DNA extraction efficiency is assumed to be 100%.). The LOD for *B. anthracis* was 2 × 10^3^ CFU/mL (appropriately 50 CFU/reaction). The LOD for *B. anthracis* solution is one log_10_ higher than the other four bacteria, which is in accordance with the above tests with pure DNA as the template.

### Absolute quantification of the bacterial cell amount by droplet digital polymerase chain reaction

Based on the copy number/reaction acquired by ddPCR, a quantification study of the bacterial population in the above PBS solutions and soil samples was also performed (Tables 2, 3). The estimated bacteria amount was consistent with the plate counts for *Y. pestis*, *B. pseudomallei*, and *Brucella* spp. Limit of quantifications (LOQ) of these three targets were all 2 × 10^3^ CFU/mL, higher than the corresponding LOD values (2 × 10^2^ CFU/mL). However, for *B. anthracis* samples, the ddPCR underestimated the bacteria population, around threefold (pure bacterial solutions) and ten-fold (soil samples) lower than the plate counts. This may be largely due to the poor DNA extraction efficiency for *B. anthracis*, which are gram-positive cocci with thick cell walls, whereas the other four target bacteria are gram-negative. For *F. tularensis*, the ddPCR highly overestimated the bacteria population, around 10-fold higher than the plate counts. This result is similar to a previous study (Ricchi et al., 2017): a single-target ddPCR assay for *F. tularensis* (with the same ddPCR system, QX200) also overestimated (around 10-fold) the bacteria amount compared to the plate-counting method.

**TABLE 2:** Estimated bacterial amount of the five selected agents in PBS buffer (CFU/mL) by ddPCR.

Amount by plate count	*^a^*Estimated bacteria amount in PBS buffer (CFU/mL)				
	
	*Y. pestis*	*B. pseudomallei*	*B. anthracis*	*Brucella* spp.	*F. tularensis*
10^6^	(1.2 ± 0.1) × 10^6^	(3.5 ± 0.3) × 10^6^	(9.2 ± 0.1) × 10^5^	(3.1 ± 0.3) × 10^6^	(7.6 ± 0.5) × 10^6^
10^5^	(1.2 ± 0.2) × 10^5^	(2.3 ± 0.1) × 10^5^	(7.8 ± 0.2) × 10^4^	(3.3 ± 0.3) × 10^5^	(1.6 ± 0.1) × 10^6^
10^4^	(1.5 ± 0.2) × 10^4^	(3.8 ± 0.5) × 10^4^	(5.0 ± 0.6) × 10^3^	(3.5 ± 0.3) × 10^4^	(2.2 ± 0.2) × 10^5^
10^3^	(2.3 ± 0.2) × 10^3^	(4.3 ± 1.0) × 10^3^	(5.3 ± 2.4) × 10^2^	(2.5 ± 1.1) × 10^3^	(1.9 ± 0.1) × 10^4^
10^2^	(1.2 ± 0.2) × 10^3^	(1.1 ± 0.3) × 10^3^	0	(4.5 ± 1.2) × 10^2^	(2.8 ± 0.2) × 10^3^

^a^The estimated bacterial amount = ddPCR result (copies/reaction) × 40. Data are presented as mean ± standard deviation, n = 3.

**TABLE 3:** Estimated bacterial amount of the five selected agents in soil samples (CFU/100 mg soil) by ddPCR.

Amount by plate count	*^a^*Estimated bacteria amount in soil (CFU/100 mg soil)				
	
	*Y. pestis*	*B. pseudomallei*	*B. anthracis*	*Brucella* spp.	*F. tularensis*
10^6^	(1.3 ± 0.1) × 10^6^	(8.8 ± 0.3) × 10^5^	(1.6 ± 0.1) × 10^5^	(4.0 ± 0.1) × 10^6^	*^b^*9.1 × 10^6^
10^5^	(8.1 ± 0.3) × 10^4^	(1.2 ± 0.1) × 10^5^	(1.6 ± 0.1) × 10^4^	(4.0 ± 0.2) × 10^5^	(2.1 ± 0.1) × 10^6^
10^4^	(9.1 ± 0.8) × 10^3^	(1.1 ± 0.1) × 10^4^	(1.4 ± 0.4) × 10^3^	(3.8 ± 0.3) × 10^4^	(1.6 ± 0.1) × 10^5^
10^3^	(1.7 ± 0.2) × 10^3^	(1.8 ± 0.2) × 10^3^	(3.1 ± 0.1) × 10^2^	(4.7 ± 0.8) × 10^3^	(1.8 ± 0.1) × 10^4^
10^2^	(4.1 ± 1.3) × 10^2^	(5.2 ± 0.8) × 10^2^	0	(8.0 ± 1.1) × 10^2^	(2.7 ± 0.5) × 10^3^

^a^The estimated bacterial amount = ddPCR result (copies/reaction) × 40.^b^Two of the three measurements exceeded the upper limit of quantitation. Other data are presented as mean ± standard deviation, n = 3.

### Performances of the droplet digital polymerase chain reaction and real-time polymerase chain reaction method in detecting spiked soil samples

When 50 simulated soil samples were tested, all the 35 positive samples (including single-target or multi-target samples at concentrations of 2 × 10^3^–2 × 10^4^ CFU/100 mg soil) could be identified by the multiple ddPCR, showing a high sensitivity (100%, 35/35) and specificity (100%, 15/15) (Supplementary Table 2). The single-target qPCR method has a sensitivity of only 65.71% (23/35), mainly due to the failure to detect samples spiked with *Y. pestis*, *B. pseudomallei*, and *B. anthracis* at the concentration of 2 × 10^3^ CFU/100 mg (Supplementary Table 3). The results show that the developed multiplex ddPCR method has higher detection sensitivity (10^2^–10^3^ CFU/100 mg soil) than the qPCR method (10^3^–10^4^ CFU/100 mg soil), and it can be reliably applied for detecting the five bacterial biothreats in suspicious soil samples.

## Discussion

The rapid multiplex assay effectively improves the detection efficiency of biothreat agents in suspicious samples. For the detection of biothreat agents, various multiplex qPCR-based methods have been developed, such as the FilmArray Biothreat Panel (Biomerieux, France) (Seiner et al., 2013), Taqman Array Card (TAC) (Thermo Fisher Scientific, United States) (Rachwal et al., 2012), and the GeneXpert system (Ceipheid, United States) (Banada et al., 2019). In contrast, there are few studies on the multiplex detection of biothreat agents by ddPCR. This study developed a 5-plex ddPCR assay for five selected bacterial biothreat agents with a two-color ddPCR system. Table 4 compares the detection performance between previous multiplex qPCR and this assay. Our method’s sensitivity is comparable to that of the FilmArray Biothreat Panel and the TAC assay. The GeneXpert kit showed the highest sensitivity of less than 10 CFU/mL for *Y. pestis*, *B. anthracis*, and *F. tularensis*, which could be due to its targets being multicopy genes on the chromosome or multicopy plasmids (Banada et al., 2019). The above qPCR methods are performed with a highly automated or integrated system, which takes obvious advantages over the current ddPCR method in operational convenience. However, they can only analyze limited sample numbers (1–8) per pouch, cartridge, or card, whereas common ddPCR can amplify and analyze 96 samples simultaneously. Therefore, the ddPCR method could be a better choice when screening many suspicious samples.

**TABLE 4:** Comparisons between reported multiplex real-time PCR and the 5-plex droplet digital PCR assay for the five selected pathogens.

Multiplex PCR	Principle	LODs (CFU/mL) and the target gene on chromosome (chr) or plasmid					References
		
		*Y. pestis*	*B. anthracis*	*F. tularensis*	*Brucella* spp.	*B. pseudomallei*	
TAC (qPCR)	Array-based multiplex	10^3^ (chr, plasmid)	10^3^ (plasmid)	10^3^ (chr)	*^a^* NM	0.5 pg/test (chr)	Rachwal et al., 2012; Weller et al., 2012
Filmarray (qPCR)	Array-based multiplex	10^3^ (plasmid)	10^2^ (plasmid)	10^3^ (chr)	NM	NM	Weller et al., 2012
GeneXpert (qPCR)	4-color channels	4.5 (plasmid)	10 (plasmid)	8.5 (*^b^*chr)	NM	NM	Banada et al., 2019
5-plex ddPCR	2-color channels	10^2^ (chr)	10^3^ (chr)	10^2^ (chr)	10^2^ (chr)	10^2^ (chr)	This study

^a^NM represents not mentioned.^b^The assay was targeted the multicopy gene ISFtu on the chromosome of F. tularensis.LOD, limit of detection; CFU, colony-forming unit; qPCR, real-time PCR; ddPCR, droplet digital PCR.

Another advantage of the ddPCR method is that it can perform quantitative analysis without relying on a standard curve. Our results showed that the estimated bacterial amount by ddPCR was consistent with the plate counting for *Y. pestis*, *B. pseudomallei*, *Brucella* spp., and *B. anthracis*. However, it overestimated the amount of *F. tularensis* samples by appropriately one log_10_, similar to a previous study (Ricchi et al., 2017). According to the study, quantitative analyses of *F. tularensis*, *Mycobacterium avium* subsp. *Paratuberculosis*, and *Listeria monocytogenes* solutions were performed by the ddPCR method, which showed the estimated number of *F. tularensis* and *M. avium* subsp. *Paratuberculosis* was one log_10_ and two log_10_ more than the plate-counting number, respectively, whereas the estimated number of *Listeria monocytogenes* was the same with the culture method. Compared with the other bacteria, *F. tularensis*, and *M. avium* subsp. *Paratuberculosis* is a strain requiring special nutrition and a long time to grow (at least 4 weeks for *M. avium*) (Pooley et al., 2016; Buse et al., 2020). We presume that the number of dead bacteria and cell-free DNA in the medium increased with the culture time, resulting in the over-estimation of bacteria number by ddPCR methods. In contrast, for those fast-growing strains, this factor made less difference between the quantifications by the ddPCR method and the plate-counting method.

In the study, the established multiplex ddPCR assay mainly focused on the genes (single-copy genes) on the chromosomal rather than the plasmid genes of the five bacteria. One consideration is the possible absence of plasmids in some strains (Marston et al., 2005; Seiner et al., 2013), and another is the limited multiplexing capability of the two-color ddPCR system. Three different targets can currently be well detected in one fluorescence channel (Leong et al., 2020) based on the amplitude multiplexing approach. However, setting more targets in one channel may reduce the difference between fluorescent clusters, making it difficult to distinguish these targets. The multiplexing capacity can be further improved using the probe-mixing approach. Note that the probe concentrations and ratios must be designed elaborately, especially when performing simultaneous detection of multiple targets. With the advent of a multicolor ddPCR system (5–6 channels), its multiplexing capacity could be considerably elevated using the above approaches. Furthermore, if ddPCR systems could increase automation and integration, efficiency and convenience would be improved.

Although the developed ddPCR assay showed a good specificity for the detection of 25 other related bacteria species, *in silico* analysis revealed that it cannot distinguish species between *F. tularensis* and *Francisella novicida* (also known as *F. tularensis* subsp. *novicida*). *F. novicida* is an environmental species that could be found in natural water and soil samples, and it has 97.7% similarity with *F. tularensis* at the genome level (Larsson et al., 2009). Consequently, most PCR-based methods could not distinguish the two species (Versage et al., 2003; Hennebique et al., 2020), and neither did our ddPCR method, which calls for a more accurate assay.

## Conclusion

With a two-color ddPCR system, a multiplex ddPCR-based method for the detection of five selected biothreat pathogens (*Y. pestis*, *B. pseudomallei*, *B. anthracis*, *Brucella* spp., and *F. tularensis*) was successfully established in this study. A multiplex assay demonstrated low detection limits (0.1–1.0 pg/μL) and high specificity for the five species. It also exhibited strong tolerances to soil samples with lower LODs (2 × 10^2^ –2 × 10^3^ CFU/100 mg soil, 5–50 CFU/reaction) than those of the qPCR method. The assay could also simultaneously detect multiple targets in one sample, providing a new multiplexing method for rapid and sensitive detection of biothreat pathogens.

## Data availability statement

The original contributions presented in this study are included in the article/Supplementary material, further inquiries can be directed to the corresponding author/s.

## Author contributions

YD performed the experiments and analyzed the data. ZY, YT, KS, ZS, and JJ performed the qPCR analysis. PZ, RY, and LX participated in interpretation of the data and critical revisions of the manuscript. YS, ZD, and YZ designed the experiments and contributed to manuscript preparation. All authors contributed to the article and approved the submitted version.

## Supplementary material

SUPPLEMENTARY FIGURE 1 Specificity of the multiplex ddPCR. Detection results of 25 species of closely related bacteria or common pathogenic bacteria with detected targets. The purple line indicates the threshold for positive signals. NC refers to the negative control (DNAase-free water).
