# Supplementary material for: Development and evaluation of a multiplex droplet digital polymerase chain reaction method for simultaneous detection of five biothreat pathogens
Source: Front Microbiol. 2022 Jul 28;13:970973. doi: 10.3389/fmicb.2022.970973 (PMC9366144; doi:10.3389/fmicb.2022.970973)
Supplement: Supplementary file 1 [file Data_Sheet_1.docx]

Supplementary Tables

**Supplementary Table 1.** Sample information of the 50 spiked soil samples.

| Sample  No. | *Y. pestis* | *B. pseudomallei* | *B. anthracis* | *Brucella spp.* | *F. tularensis* |
| --- | --- | --- | --- | --- | --- |
| 1 | 10^4^ CFU/mL | N | N | N | N |
| 2 | 10^4^ CFU/mL | 10^4^ CFU/mL | N | N | N |
| 3 | 10^4^ CFU/mL | N | N | N | N |
| 4 | 10^4^ CFU/mL | N | N | 10^4^ CFU/mL | 10^4^ CFU/mL |
| 5 | N | N | N | 10^4^ CFU/mL | 10^4^ CFU/mL |
| 6 | N | N | N | N | N |
| 7 | N | N | N | N | N |
| 8 | N | N | N | N | N |
| 9 | N | N | N | N | N |
| 10 | N | N | N | 10^4^ CFU/mL | N |
| 11 | 10^4^ CFU/mL | N | 10^4^ CFU/mL | 10^4^ CFU/mL | N |
| 12 | N | N | N | 10^4^ CFU/mL | N |
| 13 | N | N | N | N | N |
| 14 | N | 10^4^ CFU/mL | 10^4^ CFU/mL | N | 10^4^ CFU/mL |
| 15 | N | 10^4^ CFU/mL | N | N | N |
| 16 | N | N | N | N | 10^4^ CFU/mL |
| 17 | N | N | 10^4^ CFU/mL | 10^4^ CFU/mL | 10^4^ CFU/mL |
| 18 | N | N | N | N | N |
| 19 | N | N | N | N | N |
| 20 | N | N | N | N | 10^4^ CFU/mL |
| 21 | 10^4^ CFU/mL | 10^4^ CFU/mL | N | N | N |
| 22 | N | N | 10^4^ CFU/mL | N | N |
| 23 | 10^4^ CFU/mL | 10^4^ CFU/mL | N | 10^4^ CFU/mL | N |
| 24 | N | N | 10^4^ CFU/mL | N | N |
| 25 | N | 10^4^ CFU/mL | N | N | N |
| 26 | 10^3^ CFU/mL | N | N | N | N |
| 27 | 10^3^ CFU/mL | N | N | N | N |
| 28 | N | N | N | N | N |
| 29 | 10^3^ CFU/mL | N | N | 10^3^ CFU/mL | 10^3^ CFU/mL |
| 30 | N | N | N | 10^3^ CFU/mL | 10^3^ CFU/mL |
| 31 | N | N | N | N | N |
| 32 | N | N | N | N | N |
| 33 | N | N | N | N | N |
| 34 | 10^3^ CFU/mL | N | 10^3^ CFU/mL | 10^3^ CFU/mL | N |
| 35 | N | N | N | 10^3^ CFU/mL | N |
| 36 | N | N | N | N | N |
| 37 | N | 10^3^ CFU/mL | 10^3^ CFU/mL | N | 10^3^ CFU/mL |
| 38 | 10^3^ CFU/mL | N | N | 10^3^ CFU/mL | N |
| 39 | N | N | N | N | 10^3^ CFU/mL |
| 40 | N | N | 10^3^ CFU/mL | 10^3^ CFU/mL | 10^3^ CFU/mL |
| 41 | N | N | N | N | N |
| 42 | N | N | N | N | N |
| 43 | N | N | N | N | N |
| 44 | N | N | N | N | N |
| 45 | N | N | N | N | 10^3^ CFU/mL |
| 46 | 10^3^ CFU/mL | 10^3^ CFU/mL | N | N | N |
| 47 | N | N | 10^3^ CFU/mL | N | N |
| 48 | N | 10^3^ CFU/mL | N | N | 10^3^ CFU/mL |
| 49 | 10^3^ CFU/mL | 10^3^ CFU/mL | N | 10^3^ CFU/mL | N |
| 50 | N | N | 10^3^ CFU/mL | N | N |

N: the target bacteria is not spiked in the sample.**Supplementary Table 2.** Detection results for the 50 spiked soil samples by the ddPCR assay.

| Sample  No. | Detection results (Copies/reaction) | | | | |
| --- | --- | --- | --- | --- | --- |
|  | *Y. pestis* | *B. pseudomallei* | *B. anthracis* | *Brucella spp.* | *F. tularensis* |
| 1 | P (197.32) | N (0) | N (0) | N (0) | N (0) |
| 2 | P (236.83) | N (0) | P (72.83) | N (0) | N (0) |
| 3 | P (214.6) | N (0) | N (0) | N (0) | N (0) |
| 4 | P (167.28) | N (0) | N (0) | P (2015.29) | P (3272.95) |
| 5 | N (0) | N (0) | N (0) | P (1770.23) | P (2999.14) |
| 6 | N (2.55) | N (0) | N (0) | N (0) | N (0) |
| 7 | N (0) | N (0) | N (0) | N (0) | N (0) |
| 8 | N (0) | N (0) | N (0) | N (0) | N (0) |
| 9 | N (0) | N (0) | N (0) | P (1412.33) | N (0) |
| 10 | P (179.79) | N (0) | P (52.35) | P (2061.83) | N (0) |
| 11 | N (0) | N (0) | N (0) | P (1306.98) | N (0) |
| 12 | 5.05(-) | N (0) | N (0) | N (0) | N (0) |
| 13 | N (0) | P (192.78) | P (50.72) | N (0) | P (1794.43) |
| 14 | P (158.92) | N (0) | P (1794.43) | N (0) | N (0) |
| 15 | N (0) | P (90.36) | N (0) | N (0) | N (0) |
| 16 | N (0) | N (0) | N (0) | N (0) | P (1059.51) |
| 17 | N (0) | N (0) | P (29.55) | P (2298.76) | P (3696.08) |
| 18 | N (0) | N (0) | N (0) | N (0) | N (0) |
| 19 | N (2.47) | N (0) | N (0) | N (0) | N (0) |
| 20 | N (0) | N (0) | N (0) | N (0) | P (996.86) |
| 21 | P (111.87) | P (222.70) | N (0) | N (0) | N (0) |
| 22 | N (0) | N (0) | P (222.20) | N (0) | N (0) |
| 23 | P (149.32) | P (255.55) | N (0) | P (2768.56) | N (0) |
| 24 | N (0) | N (0) | P (237.64) | N (0) | N (0) |
| 25 | N (0) | P(103.58) | N (0) | N (0) | N (0) |
| 26 | P(15.93) | N (0) | N (0) | N (0) | N (0) |
| 27 | P(13.54) | N (0) | N (0) | N (0) | N (0) |
| 28 | N (5.31) | N (0) | N (0) | N (0) | N (0) |
| 29 | P (8.41) | N (0) | N (0) | P (114.61) | P (233.17) |
| 30 | N (0) | N (0) | N (0) | P (122.27) | P (187.10) |
| 31 | N (2.59) | N (0) | N (0) | N (0) | N (0) |
| 32 | N (1.32) | N (0) | N (0) | N (0) | N (0) |
| 33 | N (2.7) | N (0) | N (0) | N (1.35) | N (0) |
| 34 | P (16.44) | N (0) | P (20.56) | P (124.99) | N (0) |
| 35 | N (0) | N (0) | N (0) | P (156.25) | N (0) |
| 36 | N (2.55) | N (0) | N (0) | N (0) | N (0) |
| 37 | N (4.81) | P (28.88) | P (17.64) | N (0) | P (207.75) |
| 38 | P (17.82) | N (0) | N (0) | P (63.13) | N (0) |
| 39 | N (0) | N (0) | N (0) | N (0) | P (529.22) |
| 40 | N (0) | N (0) | P (17.41) | P (111.37) | P (193.56) |
| 41 | N (1.29) | N (0) | N (0) | N (0) | N (0) |
| 42 | N (0) | N (0) | N (0) | N (0) | N (0) |
| 43 | N (0) | N (0) | N (0) | N (0) | N (0) |
| 44 | N (2.54) | N (0) | N (0) | N (0) | N (0) |
| 45 | N (0) | N (0) | N (0) | N (0) | P (444.70) |
| 46 | P (6.44) | P (15.45) | N (0) | N (0) | N (0) |
| 47 | N (3.84) | N (0) | P (29.44) | N (0) | N (0) |
| 48 | N (0) | P (22.47) | N (0) | N (0) | P (160.28) |
| 49 | P (9.19) | P (11.81) | N (0) | P (92.04) | N (0) |
| 50 | N (0) | N (0) | P (36.24) | N (0) | N (0) |

“N” represents negative result; “P” represents positive result.

**Supplementary Table 3.** Detection results for the 50 spiked soil samples by the single-target qPCR method.

| Sample No. | Detection results (Ct values / the bacteria concentration) | | | | |  |
| --- | --- | --- | --- | --- | --- | --- |
|  | *Y. pestis* | *B. pseudomallei* | *B. anthracis* | *Brucella spp.* | *F. tularensis* | |
| 1 | P (29.05) | N | N | N | N | |
| 2 | P (29.16) | N | P (33.06) | N | N | |
| 3 | P (29.13) | N | N | N | N | |
| 4 | P (29.56) | N | N | P (28.06) | P (27.42) | |
| 5 | N | N | N | P (28.04) | P (27.51) | |
| 6 | N | N | N | N | N | |
| 7 | N | N | N | N | N | |
| 8 | N | N | N | N | N | |
| 9 | N | N | N | P (27.89) | N | |
| 10 | P (29.05) | N | P (32.63) | P (27.44) | N | |
| 11 | N | N | N | P (28.10) | N | |
| 12 | N | N | N | N | N | |
| 13 | N | P (29.56) | P (32.22) | N | P (26.42) | |
| 14 | P (29.43) | N | N | P (27.47) | N | |
| 15 | N | N | N | N | N | |
| 16 | N | N | N | N | P (28.88) | |
| 17 | N | N | P (32.69) | P (26.95) | P (26.55) | |
| 18 | N | N | N | N | N | |
| 19 | N | N | N | N | N | |
| 20 | N | N | N | N | P (28.95) | |
| 21 | P (29.64) | P (30.15) | N | N | N | |
| 22 | N | N | P (30.29) | N | N | |
| 23 | P (29.53) | P (29.66) | N | P (26.80) | N | |
| 24 | N | N | P (30.23) | N | N | |
| 25 | N | P (30.88) | N | N | N | |
| 26 | FN (10^3^ CFU/mL) | N | N | N | N | |
| 27 | FN (10^3^ CFU/mL) | N | N | N | N | |
| 28 | N | N | N | N | N | |
| 29 | FN (10^3^ CFU/mL) | N | N | P (32.68) | P (31.28) | |
| 30 | N | N | N | P (32.73) | P (31.30) | |
| 31 | N | N | N | N | N | |
| 32 | N | N | N | N | N | |
| 33 | N | N | N | N | N | |
| 34 | FN (10^3^ CFU/mL) | N | FN (10^3^ CFU/mL) | P (31.27) | N | |
| 35 | N | N | N | P (32.08) | N | |
| 36 | N | N | N | N | N | |
| 37 | N | FN (10^3^ CFU/mL) | FN (10^3^ CFU/mL) | N | P (31.13) | |
| 38 | FN (10^3^ CFU/mL) | N | N | P (32.97) | N | |
| 39 | N | N | N | N | P (30.11) | |
| 40 | N | N | FN (10^3^ CFU/mL) | P (32.22) | P (31.48) | |
| 41 | N | N | N | N | N | |
| 42 | N | N | N | N | N | |
| 43 | N | N | N | N | N | |
| 44 | N | N | N | N | N | |
| 45 | N | N | N | N | P (30.12) | |
| 46 | FN (10^3^ CFU/mL) | FN (10^3^ CFU/mL) | N | N | N | |
| 47 | N | N | FN (10^3^ CFU/mL) | N | N | |
| 48 | N | FN (10^3^ CFU/mL) | N | N | P (30.78) | |
| 49 | FN (10^3^ CFU/mL) | FN (10^3^ CFU/mL) | N | P (32.23) | N | |
| 50 | N | N | FN (10^3^ CFU/mL) | N | N | |

“N” represents negative result; “P” represents positive result; “FN” represents false negative results.

The sample No. with underlines represents that it contains target bacteria undetected by the qPCR method.
