# Supplementary figures and images for: Development and evaluation of a multiplex droplet digital polymerase chain reaction method for simultaneous detection of five biothreat pathogens
Source: Front Microbiol. 2022 Jul 28;13:970973. doi: 10.3389/fmicb.2022.970973 (PMC9366144; doi:10.3389/fmicb.2022.970973)

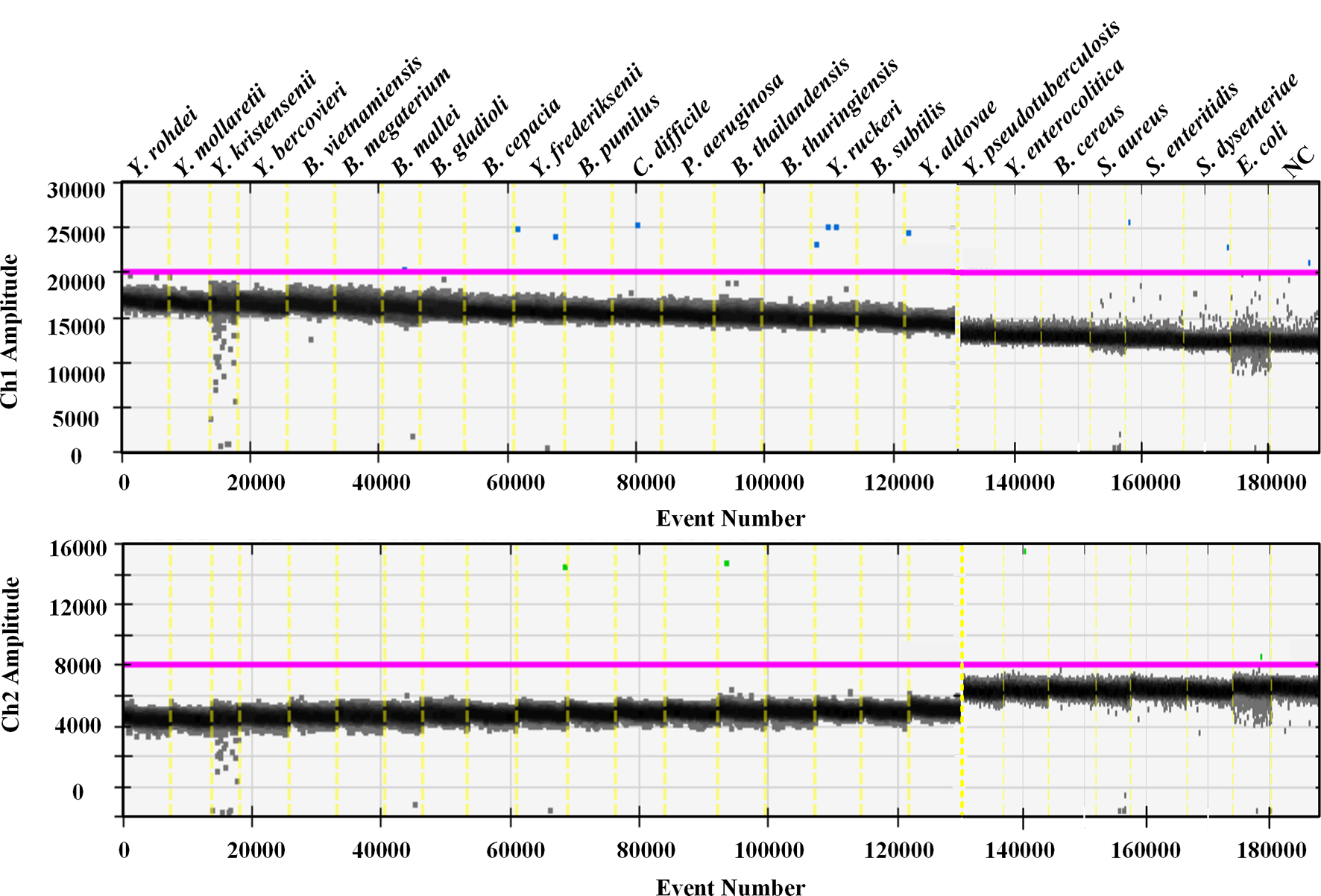

Supplement: SUPPLEMENTARY FIGURE 1 — Specificity of the multiplex ddPCR. Detection results of 25 species of closely related bacteria or common pathogenic bacteria with detected targets. The purple line indicates the threshold for positive signals. NC refers to the negative control (DNAase-free water). [file Image_1.TIF]
